# Bacterial Profile, Multi-Drug Resistance and Seasonality Following Lower Limb Orthopaedic Surgery in Tropical and Subtropical Australian Hospitals: An Epidemiological Cohort Study

**DOI:** 10.3390/ijerph17020657

**Published:** 2020-01-20

**Authors:** Mark L. Vickers, Emma L. Ballard, Patrick N. A. Harris, Luke D. Knibbs, Anjali Jaiprakash, Joel M. Dulhunty, Ross W. Crawford, Benjamin Parkinson

**Affiliations:** 1 Biomedical Engineering and Clinical Sciences, Queensland University of Technology, Brisbane 4000, Australia; r.crawford@qut.edu.au; 2 QIMR Berghofer Medical Research Institute, Brisbane 4006, Australia; emma.ballard@qimrberghofer.edu.au; 3 UQ Centre for Clinical Research, The University of Queensland, Brisbane 4006, Australia; p.harris@uq.edu.au; 4 School of Public Health, The University of Queensland, Brisbane 4006, Australia; l.knibbs@uq.edu.au; 5 Science and Engineering Faculty, Queensland University of Technology, Brisbane 4000, Australia; anjali.jaiprakash@qut.edu.au; 6 School of Public Health and Social Work, Queensland University of Technology, Brisbane 4006, Australia; Joel.Dulhunty@health.qld.gov.au; 7 Department of Orthopaedics, Cairns Base Hospital, Cairns 4870, Australia; benjaminparkinson@gmail.com

**Keywords:** orthopaedics, multiple drug resistance, bacteria, antibacterial agents, epidemiology

## Abstract

We aimed to describe the epidemiology, multi-drug resistance and seasonal distribution of bacteria cultured within 12 months following lower limb orthopaedic surgery in tropical and subtropical Australian hospitals between 2010 and 2017. We collected data from four tropical and two subtropical hospitals. Categorical variables were examined using the Pearson Chi-squared test or Fisher’s Exact test, and continuous variables with the Student t-test or Mann–Whitney U test. A Poisson regression model was used to examine the relationship between season, weather and the incidence of Staphylococcus and nonfermentative species. We found that at tropical sites, nonfermenters (*Pseudomonas aeruginosa* and *Acinetobacter baumannii*) were more common (28.7% vs. 21.6%, *p* = 0.018), and patients were more likely to culture multi-drug-resistant (MDR) nonfermenters (11.4% vs. 1.3%, *p* = 0.009) and MDR *Staphylococcus aureus* (35.9% vs. 24.6%, *p* = 0.006). At tropical sites, patients were more likely to be younger (65.9 years vs. 72.0, *p* = < 0.001), male (57.7% vs. 47.8%, *p* = 0.005), having knee surgery (45.3% vs. 34.5%, *p* = 0.002) and undergoing primary procedures (85.0% vs. 73.0%, *p* = < 0.001). Species were similar between seasons in both tropical and subtropical hospitals. Overall, we found that following lower limb orthopaedic surgery in tropical compared with subtropical Australia, patients were more likely to culture nonfermenters and some MDR species.

## 1. Introduction

It is well known that Gram-positive organisms including *Staphylococcus aureus* and coagulase-negative staphylococci (CONS) are the most frequently identified pathogens complicating orthopaedic surgery [1]. However, Gram-negatives including *Pseudomonas aeruginosa*, *Klebsiella pneumoniae* and *Acinetobacter baumannii* are also relatively common, involved in up to 17% of infections [1,2,3]. Following the recent era of widespread antibiotic use, a number of bacteria have come to exhibit high levels of antibiotic resistance, and it is increasingly recognized that infection with a multi-drug-resistant (MDR) organism increases morbidity, mortality and healthcare costs [4,5,6]. While local bacterial profile and resistance rates are influenced by a range of factors, including antibiotic use and host immune status, several orthopaedic studies have also suggested that environment and season influence bacterial pathogenicity due to changes in human activity and weather [7,8,9,10,11,12,13]. At higher temperatures, it has been shown that there are increased levels of bacteria present in some anatomical locations, potentially due to changes in skin moisture and pH [13,14].

Though conditions within the orthopaedic operating theatre are controlled, patients are soon discharged to the community, where weather conditions may be ideal for bacterial growth. In Australia, hospitals in tropical regions experience annual cycles of high temperature and high relative humidity. In their study of national joint replacement registry data, Parkinson et al. recently identified higher early revision rates for prosthetic joint infection (PJI) in tropical vs. subtropical Australian hospitals [9]. At their tropical Australian institution, Armit et al. also suggested that relative humidity played an important role in postoperative infection rates [15]. To our knowledge, there are no prior studies that describe the epidemiology or multi-drug resistance of bacteria following orthopaedic surgery in a tropical region [13,16]. A better understanding of the bacteria driving regional orthopaedic infections will enhance surveillance, inform antibiotic selection and assist infection control.

Hence, the objectives of this study were to describe the bacterial species cultured from swabs taken following hip and knee surgery between 2010 and 2017 in northern Australian hospitals; identify differences in bacterial species and resistance rates between tropical and subtropical locations; and describe the prevalence of bacterial species by season.

## 2. Materials and Methods

### 2.1. Hospital and Patient Data

**Figure 1:**
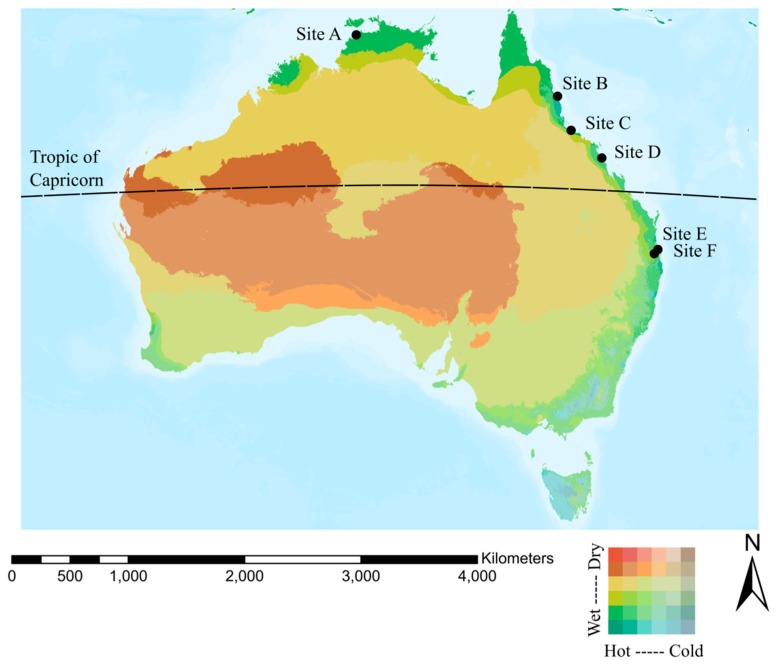
Climate map of Australia showing hospital site locations. Climate layer consists of 37 bioclimate zones derived from a combination of moisture and temperature variables, source: Esri, HERE, Garmin, FAO, NOAA, USGS, © OpenStreetMap contributors and the GIS User Community, Source: Esri; Metzger, et.al. 2012.

We conducted a retrospective, multisite, cohort study. Four tropical and two subtropical Australian public hospitals located across four geographic regions were included. These sites were chosen because they were major orthopaedic centres for the included regions. Sites were defined as tropical or subtropical based on the Kӧppen climate classification system used by the Australian Bureau of Meteorology [17]. Though within a Kӧppen subtropical region, we defined Site D as tropical because it experienced similar humidity and temperature to tropical sites, was north of the Tropic of Capricorn (Latitude 23.43673°) and bordered a tropical zone. A map showing climate regions within Australia and the location of included sites is shown in Figure 1.

We included all patients over the age of 18 who underwent any lower limb orthopaedic procedure between 1 January 2010 and 30 June 2017 and who had a positive wound swab cultured. We collected data on age, gender, procedure type, procedure dates and admission dates. For patients who received multiple procedures across the study period, we considered each procedure within its hospital admission as a discrete event, and where multiple procedures occurred during a single admission, we collapsed these into a single event. Patients were identified by searching hospital inpatient databases using the International Classification of Diseases (ICD) coding system. Our search included 78 unique ICD lower limb procedure codes. Traumatic open fractures were not included. A full list of included ICD codes and procedure descriptions are available in Appendix A.

Ethics approval for five hospitals in the state of Queensland was granted by the Townsville Hospital and Health Service Human Research and Ethics Committee (HREC/17/QTHS/55). Ethics approval for one hospital in the Northern Territory was granted by the Human Research and Ethics Committee of the Northern Territory Department of Health and Menzies School of Health Research (2017–2992).

### 2.2. Microbiological Data

Bacteria were included if collected from patients on the day of surgery or any day within the following 12 months. We collected data on species, collection date, antibiotic resistance and body site. Where a patient cultured the same species on more than one occasion, we recorded that species only once using the earliest collection date after the orthopaedic procedure. Swab results were obtained from the relevant pathology Laboratory Information System (AUSLAB, Citadel Health Pty Ltd., Melbourne, Australia; Labtrak, Intersystems, Cambridge, MA, USA).

We grouped Gram-positive bacteria into four categories: *S. aureus*, Coagulase negative staphylococci (CONS) (which included *Staphylococcus epidermidis*, *Staphylococcus capitis* and *Staphylococcus lugdunensis*), *Enterococcus* spp. and *Streptococcus* spp. For enterococci, we included *Enterococcus faecalis* and *Enterococcus faecium*. Our *Streptococcus* group comprised the following: beta-haemolytic streptococci (Lancefield group A, group B, group C and group G), *Peptostreptococcus*, *Streptococcus pneumoniae*, viridans group and anginosus-constellatus group.

Gram-negative bacteria were grouped into Enterobacteriaceae, and Gram-negative nonfermentative spp. Enterobacteriaceae included *Morganella morganii*, *Proteus mirabilis*, *Escherichia coli*, *Klebsiella* spp., *Serratia marcescens* and *Enterobacter cloacae*. Gram-negative nonfermenters included *P. aeruginosa* and *A. baumannii*.

We defined superficial and deep infection based on the time component described in the Centers for Disease Control guidelines, which limits superficial surgical site infection (SSI) as occurring within 30 days following an operation and deep SSI as occurring from 30 to 90 days [18]. We also included a late infection category, defined as occurring from 90 to 365 days. We limited this to one year in an attempt to control for potential patient exposure to multiple seasonal weather cycles, other surgery, illness or travel. Time of isolation of pathogen from surgery was used as a proxy for infection only, and patient charts were not reviewed to confirm clinician-diagnosed infection. Swab collection site was categorized as hip, knee, other or not specified. We included only bacteria obtained from superficial wounds, orthopaedic surgical wounds and a joint sinus or during orthopaedic procedures.

Multi-drug resistance was defined as nonsusceptibility to at least one agent in three or more classes, as described by the joint initiative on standard definitions for acquired resistance [19]. We automatically assigned Methicillin-resistant *S. aureus* (MRSA) and Vancomycin-resistant enterococci (VRE) as MDR.

### 2.3. Meteorological Data

For each site, we collected data across the study period for temperature, relative humidity, dew point and rainfall. Dew point is the temperature at which water vapour condenses into liquid and is useful because it is formulated with consideration of atmospheric pressure, temperature and relative humidity. Multiple weather stations within a 25 km radius of each hospital were used to calculate regional conditions for the hospital catchment.

### 2.4. Statistical Analysis

SPSS (Version 25) and R (Version 3.6.0) were used for statistical analysis. Categorical variables were examined using the Pearson Chi-squared test and Fisher’s Exact test where more than 20% of the expected values were less than 5. Continuous variables were examined using the Student t-test or Mann–Whitney U test where data were not normally distributed.

The prevalence of bacterial species across the four seasons was compared using the Pearson Chi-squared test or Fisher’s exact test. To assess the relationship between weather factors and bacterial incidence, a Poisson regression model was used to examine *Staphylococcus* and nonfermentative species separately. Count data aggregated by month and year were examined. As hospital size population numbers were inherently different between tropical and subtropical locations, models were run separately for each location and adjusted for season and weather factors (temperature, dew point, relative humidity and rainfall). Interaction terms were not included in the final models. Weather on the date of surgery was examined with separate models for average and highest value within the month. A natural cubic spline with 7 degrees of freedom was used to explore long-term trends, but was not included in the final models presented here, as the results were not significant.

## 3. Results

### 3.1. Meteorological Factors, Patient and Swab Characteristics

Tropical regions of Australia experience an annual “wet season” between November and April, which is characterised by high humidity, tropical storms and monsoonal rain. Subtropical regions of Australia do not experience this weather pattern. We found significant differences between tropical and subtropical weather. The average daily dew point for tropical sites was higher than for subtropical sites (19.3 °C ± 4.5 vs. 14.6 °C ± 4.8). Average daily relative humidity was also higher for tropical regions (73.7% ± 10.4% vs. 69.3% ± 10.0%), as was average temperature (25.3 °C ± 3.5 vs. 21.0 °C ± 4.0). Though timing of rainfall throughout the year differed, we found that the median rainfall was similar between locations with a median of 0.0 mm (interquartile range (IQR), 0.0–2.5) for tropical sites and 0.1 mm (IQR, 0.0–1.8) for subtropical sites.

We collected data on 19,107 discrete admissions involving 16,176 patients. A total of 867 (4.5%) admissions involving 841 (5.2%) patients produced a culture-positive swab in the 12 months following lower limb orthopaedic surgery. Of the 841 culture-positive patients, 817 (97.1%) had one procedure during their admission, while 22 (2.6%) had two procedures and two (0.2%) patients underwent three procedures. Details of positive swab status per site are included in Appendix B.

There was statistically significant variation in patient and swab characteristics between tropical and subtropical sites. At tropical sites, patients were younger, with a mean age at time of procedure of 65.9 years (standard deviation (SD), 16.9) vs. subtropical at 72 years (SD, 13.8, *p* = < 0.001). At tropical sites, patients were more likely to be male (57.7% vs. 47.8%, *p* = 0.005). At tropical sites, a greater proportion of surgeries were related to the knee (45.3%) vs. subtropical (34.5%, *p* = 0.002) and patients were also more likely to be undergoing primary rather than revision procedures (85.0% vs. 73.0%, *p* = < 0.001). A full description of significant characteristics by tropical and subtropical location is shown Table 1.

### 3.2. Bacterial Characteristics

A total of 867 positive bacterial swabs were identified. The majority of bacteria cultured were Gram-positive (715, 82.5%). The most common pathogen overall was *S. aureus* (527, 60.8%), followed by Nonfermenters (224, 25.8%) and Enterobacteriaceae spp. (129, 14.9%). MDR status was identified in 66.0% of CONS, 38.8% of Enterobacteriaceae, 37.3% of *Enterococcus* spp. and 31.5% of *S. aureus*. Patients who underwent surgery at tropical sites were more likely to culture MDR *S. aureus* (35.9%) vs. those at subtropical locations (24.9%, *p* = 0.006). Further details of antimicrobial sensitivity rates for MDR bacteria are shown in Table 2. We also found that *S. aureus* was more commonly identified as a late infective pathogen at tropical sites (35.0%) vs. subtropical sites (23.2%, *p* = 0.004). Overall, we found that Enterobacteriaceae were less common at tropical sites (12.9% vs. 17.8%, *p* = 0.047), and there was also a significant difference in the body region being swabbed, with a greater proportion of hip swabs culturing Enterobacteriaceae at subtropical sites (*p* = 0.038). There was a higher likelihood of culturing *A. baumannii* or *P. aeruginosa* (28.7%) at tropical sites vs. subtropical (21.6%, *p* = 0.018), and MDR nonfermenters tended to be associated with tropical locations (11.4% vs. 1.3%, *p* = 0.009). We also note that *Streptococcus* spp. differed by location, though bacterial numbers were low. Though not statistically significant, we note that polymicrobial swab status was more likely in tropical locations (22.9% vs. 17.5%, *p* = 0.055). Full details of bacterial species by tropical and subtropical location are shown in Appendix C. No significant differences were observed between seasons or months for any of the bacterial species at tropical or subtropical locations (Appendix D and Appendix E). Poisson regression models of *Staphylococcus* spp. and nonfermenters by season and month showed no evidence of seasonal spikes in bacterial count. Average and maximum values of meteorological variables were included as main effects, but none were significant in any of the models examined and thus, are not shown.

## 4. Discussion

Our study aimed to describe the epidemiology, resistance rates and seasonal distribution of bacteria cultured following lower limb orthopaedic surgery in tropical and subtropical northern Australian hospitals between 2010 and 2017. We identified several statistically significant differences between tropical and subtropical sites. At tropical sites, we found that nonfermenters (*P. aeruginosa* and *A. baumannii*) were more common and that patients were more likely to culture MDR nonfermenters and MDR *S. aureus*. At tropical sites, we also found that patients with culture-positive swab collection were more likely to be younger, male, had undergone knee surgery and have had primary procedures. These differences likely reflect variations between regional and metropolitan orthopaedic practice and the importance of understanding the regional antibiogram. Though weather factors differed between tropical and subtropical regions, there was no evidence that weather influenced bacterial count and no evidence of seasonality at either tropical or subtropical sites.

The frequency of *P. aeruginosa* and *A. baumannii* identified in our study was higher than elsewhere in the literature. In their cohort of orthopaedic trauma patients in Brazil, Tuon et al. found that *P. aeruginosa* made up 11.6% of bacteria cultured from surgical site infections, while Dudareva et al. in the United Kingdom and Ma et al. in China found that nonfermenters comprised 6.5% and 7.6% of cultured organisms from chronic osteomyelitis patients, respectively [20,21,22]. In Taiwan, Wang et al. also identified lower rates of *P. aeruginosa*, which made up just 2.8% of pathogens causing prosthetic joint infection [23]. Though the mechanisms are unclear, in our study, the higher frequency of nonfermenters identified at tropical vs. sub-tropical sites likely reflects a predilection of these pathogens for included regions, and the complexity of niches may mean that, even within tropical environments, there are regions of varying suitability for these organisms. Meteorological differences between tropical and subtropical regions may be driving the differences in pathogen profile between regions in our study. We also found that MDR nonfermenters and MDR *S. aureus* rates were higher at tropical sites vs. subtropical. Reported cases of MDR nonfermenters in the orthopaedic literature are too low to be comparable; however, we note that our rates of MDR *S. aureus* are consistent with other studies [20,21,23,24,25,26]. In their assessment of pathogens cultured from surgical site infections following joint arthroplasty and spinal fusions, Norton et al. found that 33% of *S. aureus* was methicillin-resistant and similarly, Dudareva et al. identified that 22.5% of *S. aureus* species cultured from chronic osteomyelitis patients in the United Kingdom were MDR [20,24]. MDR *S. aureus* and nonfermenters may be more endemic in the Australian tropical healthcare system for a variety of reasons. The influence of tropical weather on the skin microbiome, local hospital and community antibiotic use, human travel, agriculture and regional wastewater management strategies likely all contribute to the pathogenic potential of these MDR bacteria in the region studied [6].

We found that at tropical sites, patients with positive swabs following surgery were more likely to be younger, male, had undergone knee surgery and have had primary procedures. Age and gender have been well described in the literature as risk factors for infection following orthopaedic surgery, and it is also known from national joint replacement registry data that knee replacements pose a higher risk than for hips [27,28,29,30]. We note that both subtropical sites included in our study are located within the same metropolitan city, while the four tropical sites are regional and remote with substantially lower population densities. It is likely that the differences in patient and surgery characteristics identified in our study reflect trends in metropolitan versus regional orthopaedic practice.

We did not identify any evidence of seasonal spikes in microbiological samples positive for potentially pathogenic bacteria or any relationship between weather and frequency. This is in contrast to prior studies that have demonstrated an increased incidence of orthopaedic infections at certain times of the year [7,9,11,31]. For instance, in their United States study, Anthony et al. showed an increased risk of 30 day readmission for SSI following lower limb arthroplasty in the summer months, and following spinal surgery at their institution. Gruskay et al. also identified an increased incidence of wound infection during the summer and autumn [11,31]. Our study suggests that in tropical and subtropical Australia there is either no seasonal spike in infection rates or this is too small to be appreciable. This is supported by our recent meta-analysis, which demonstrated from cumulative data only a marginally increased risk of postoperative infection following orthopaedic surgery in the warmer months [32]. While prior studies have suggested that warmer, wetter conditions lead to increased infection risk, we believe that the main effect relates to a change in pathogen profile. It is likely that in tropical conditions, different pathogens predominate compared with temperate climates and that any increased risk of infection is due to inadequate antimicrobial prophylaxis for these species. In north-eastern Australia there does not appear to be, however, a seasonal increase in microbiologial sample counts due to this difference in pathogen profile.

Our study had several limitations. Firstly, we used the time between surgery and pathogen culture as a proxy for defining infection type. This is only one component of the CDC definition for surgical site infections, and individual charts were not reviewed to confirm infection status, as such it is likely that a number of our bacterial data points were contaminants only. We attempted to control for this by only including bacteria labelled as obtained from superficial wounds, orthopaedic surgical sites, joint sinuses and intraoperatively. We also included swab data from both the day of surgery and on any day in the 12 months following. While this was intended to capture potential infections which occur both at the time of surgery and possibly days or weeks later, we note that this data collection method may have led to detection bias. In addition, clinicians frequently treat orthopaedic surgical site infections empirically without swab collection, so it is likely that our data represents only a subset of postoperative orthopaedic pathogens. Similarly, our study included only public patients, so our findings may not be broadly transferable. Finally, our research question may have better been served by comparing temperate sites with tropical.

## 5. Conclusions

At tropical vs. subtropical Australian hospitals between 2010 and 2017, patients who cultured bacteria in the 12 months following any lower limb orthopaedic surgery were more likely to culture nonfermenters (*P. aeruginosa* and *A. baumannii*), MDR nonfermenters and MDR *S. aureus*. These patients were also more likely to be younger males undergoing knee surgery and having primary procedures. In contrast to prior studies, we identified no increase in positive swab status at warmer times of the year.

## Figures and Tables

**Table 1:** Differences between tropical versus subtropical hospitals for patient characteristics and bacteria cultured in the 12 months following lower limb orthopaedic procedures.

Characteristic	Sub-Category	Total (*n* = 867)	Tropical (*n* = 519)	Sub-Tropical (*n* = 348)	*p*
Age at procedure		68.3 (16.0)	65.9 (16.9)	72.0 (13.8)	<0.001
Gender ^1^	Female	389 (46.3%)	213 (42.3%)	176 (52.2%)	0.005
	Male	452 (53.7%)	291 (57.7%)	161 (47.8%)	
Surgical site	Hip	512 (59.1%)	284 (54.7%)	228 (65.5%)	0.002
	Knee	355 (40.9%)	235 (45.3%)	120 (34.5%)	
Procedure type	Primary	695 (80.2%)	441 (85.0%)	254 (73.0%)	<0.001
	Revision	172 (19.8%)	78 (15.0%)	94 (27.0%)	
Polymicrobial swab		180 (20.8%)	119 (22.9%)	61 (17.5%)	0.055
Number of procedures during admission (median (IQR))		1 (1 − 1)	1 (1 − 1)	1 (1 − 1)	0.50
MDR status	MDR *S. aureus*	166 (31.5%)	115 (35.9%)	54 (24.6%)	0.006
	MDR Nonfermenters (*P. aeruginosa* and *A. baumannii*)	18 (8.0%)	17 (11.4%)	1 (1.3%)	0.009
Organism count	Enterobacteriaceae	129 (14.9%)	67 (12.9%)	62 (17.8%)	0.047
	Nonfermenters (*P. aeruginosa* & *A. baumannii*)	224 (25.8%)	149 (28.7%)	75 (21.6%)	0.018
Swab collection for Staphylococcus (*n* = 527)	Superficial <30 days	236 (44.8%)	126 (39.4%)	110 (53.1%)	0.004
	Deep 30–90 days	131 (24.9%)	82 (25.6%)	49 (23.7%)	
	Late 90–365 days	160 (30.4%)	112 (35.0%)	48 (23.3%)	
Enterobacteriaceae swab site (*n* = 129)	Hip	61 (47.3%)	25 (37.3%)	36 (58.1%)	0.038
	Knee	41 (31.8%)	26 (38.8%)	15 (24.2%)	
	Other body site	10 (7.8%)	8 (11.9%)	2 (3.2%)	
	Not specified	17 (13.2%)	8 (11.9%)	9 (14.5%)	

^1^ Calculated using person units. IQR, interquartile range; MDR, multi-drug-resistant.

**Table 2:** Antibiotic susceptibilities of MDR bacteria cultured from patients in the 12 months following orthopaedic lower limb surgery in Australian tropical and subtropical hospitals.

Organism/Organism Group	Antibiotic ^1^	Percentage of MDR Isolates Susceptible
MDR *S. aureus* ^2^ (*n* = 166)	Flucloxacillin or Cephazolin	30%
	Erythromycin or Clindamycin	50%
	Trimethoprim-Sulphamethoxazole	79%
	Vancomycin	99%
MDR CONS (*n* = 31)	Flucloxacillin or Cephazolin	10%
	Erythromycin or Clindamycin	16%
	Trimethoprim-Sulphamethoxazole	48%
	Vancomycin	100%
MDR Enterococci (*n* = 22)	Ampicillin	73%
	Vancomycin	77%
	Teicoplanin	95%
MDR Enterobacteriaceae (*n* = 50)	Ampicillin	0%
	Amoxicillin-Clavulanic Acid	4%
	Cephazolin	4%
	Trimethoprim-Sulphamethoxazole	82%
	Ceftriaxone	86%
	Piperacillin-Tazobactam	88%
	Gentamicin	90%
	Ciprofloxacin	90%
	Meropenem	100%
MDR *P. aeruginosa* and *A. baumannii* (*n* = 18)	Ceftazidime	44%
	Ciprofloxacin	50%
	Piperacillin-Tazobactam	61%
	Gentamicin	67%
	Meropenem	67%
Streptococci (*n* = 0)		

^1^ Only antibiotics commonly used and of interest are listed. ^2^ Resistance profile not available for four MRSA data points. CONS, coagulase-negative staphylococci.

## Appendix A

### List of All Included Orthopaedic Procedures

**Number**	**ICD Code**	**Procedure Description**
1.	1488 50393-00	Bone graft to pelvis
2.	1488 48200-00	Bone graft to femur
3.	1488 48203-00	Bone graft to femur with internal fixation
4.	1489 47522-00	Hemi-arthroplasty of femur (Austin Moore)
5.	1489 49312-00	Excision arthroplasty of hip
6.	1489 49315-00	Partial arthroplasty of hip
7.	1489 90607-00	Resurfacing of hip, unilateral
8.	1489 90607-01	Resurfacing of hip, bilateral
9.	1489 49318-00	Total arthroplasty of hip, unilateral
10.	1489 49319-00	Total arthroplasty of hip, bilateral
11.	1490 50121-00	Transplantation of iliopsoas tendon to greater trochanter
12.	1490 50387-00	Transfer of iliopsoas tendon to greater trochanter
13.	1490 50387-01	Transfer of abdominal musculature to greater trochanter
14.	1490 50387-02	Transfer of adductors to ischium
15.	1491 49300-00	Arthrodesis of sacro-iliac joint
16.	1491 49306-00	Arthrodesis of hip
17.	1491 48500-00	Epiphysiodesis of femur
18.	1491 48506-00	Epiphysiodesis of femur and tibia and fibula
19.	1491 50224-09	En bloc resection of lesion of soft tissue involving penis (prosthesis)
20.	1491 50224-10	En bloc resection of lesion of soft tissue involving penis (allograft)
21.	1491 50224-11	En bloc resection of lesion of soft tissue involving penis (autograft)
22.	1491 96225-00	Arthroscopic repair of hip
23.	1491 90552-00	Other repair of hip
24.	1511 49561-02	Arthroscopic removal of loose body of knee with debridement/osteoplasty/chondroplasty
25.	1511 49562-02	Arthroscopic removal of loose body of knee with chondroplasty/drilling/implant
26.	1512 49509-01	Arthrodesis of knee
27.	1512 49512-00	Arthrodesis of knee with removal of prosthesis
28.	1513 48206-00	Bone graft to tibia
29.	1513 48209-00	Bone graft to tibia with internal fixation
30.	1514 49561-00	Arthroscopic lateral release of knee with debridement/osteoplasty/chondroplasty
31.	1514 49562-00	Arthroscopic lateral release of knee with chondroplasty/drilling/implant
32.	1515 50411-00	Resection of distal femur and proximal tibia with knee fusion
33.	1515 50414-00	Resection of distal femur and proximal tibia with knee fusion and rotationplasty
34.	1516 50357-00	Transfer of rectus femoris hamstring tendon
35.	1516 50357-01	Transfer of medial hamstring tendon
36.	1516 50357-02	Transfer of lateral hamstring tendon
37.	1516 50360-00	Transfer of combined medial and lateral hamstring tendon
38.	1516 49503-03	Transfer of tendon or ligament of knee (not elsewhere defined)
39.	1516 50423-00	Transfer of fibula to tibia with external fixation
40.	1517 49561-01	Arthroscopic menisectomy of knee with debridement, osteoplasty or chondroplasty
41.	1517 49562-01	Arthroscopic menisectomy of knee with chondroplasty/drilling/implant
42.	1518 49517-00	Hemi-arthroplasty of knee
43.	1518 49518-00	Total arthroplasty of knee, unilateral
44.	1518 49519-00	Total arthroplasty of knee, bilateral
45.	1518 49534-01	Total replacement arthroplasty of patellofemoral joint of knee
46.	1519 49521-00	Total arthroplasty of knee with bone graft to femur, unilateral
47.	1519 49521-01	Total arthroplasty of knee with bone graft to femur, bilateral
48.	1519 49521-02	Total arthroplasty of knee with bone graft to tibia, unilateral
49.	1519 49521-03	Total arthroplasty of knee with bone graft to tibia, bilateral
50.	1519 49524-00	Total arthroplasty of knee with bone graft to femur and tibia, unilateral
51.	1519 49524-01	Total arthroplasty of knee with bone graft to femur and tibia, bilateral
52.	1492 49346-00	Revision of partial arthroplasty of hip
53.	1492 49324-00	Revision of total arthroplasty of hip
54.	1492 49327-00	Revision of total arthroplasty of hip with bone graft to acetabulum
55.	1492 49330-00	Revision of total arthroplasty of hip with bone graft to femur
56.	1492 49333-00	Revision of total arthroplasty of hip with bone graft to acetabulum and femur
57.	1492 49339-00	Revision of total arthroplasty of hip with anatomic specific allograft to acetabulum
58.	1492 49342-00	Revision of total arthroplasty of hip with anatomic specific allograft to femur
59.	1492 49345-00	Revision of total arthroplasty of hip with anatomic specific allograft to acetabulum and femur Internal
60.	1521 47588-00	Fixation of intra-articular fracture of femoral condyle with repair
61.	1521 47588-01	Internal fixation of intra-articular fracture of tibial articular surface of knee with repair
62.	1521 47591-00	Internal fixation of intra-articular fracture of femoral condyle and tibial articular surface
63.	1522 49539-00	Arthroscopic reconstruction of knee
64.	1522 49539-01	Reconstruction of knee
65.	1522 49542-00	Arthroscopic reconstruction of cruciate ligament of knee and repair meniscus
66.	1522 49542-01	Reconstruction of cruciate ligament of knee with repair of meniscus
67.	1522 90611-00	Patellar tendon shortening
68.	1522 90611-01	Patellar tendon advancement
69.	1522 50417-00	Reconstruction of knee involving transfer of fibula and tibia or repair of quads
70.	1523 49530-00	Revision of total arthroplasty of knee with bone graft to femur
71.	1523 49530-01	Revision of total arthroplasty of knee with bone graft to tibia
72.	1523 49533-00	Revision of total arthroplasty of knee with bone graft to femur and tibia
73.	1523 49554-00	Revision of total arthroplasty of knee with anatomic specific allograft
74.	1524 49545-00	Revision of arthrodesis of knee
75.	1524 49548-00	Revision of patellofemoral stabilisation of knee
76.	1524 49551-00	Revision of reconstructive surgery of knee
77.	1524 49527-00	Revision of total arthroplasty of knee
78.	1524 90562-00	Patella resurfacing

## Appendix B

### Admission, Swab and Weather Station Data by Hospital Site

**Status**	**Hospital**	**Location Latitude and Longitude**	**Number of Weather Stations Within 25 km Radius**	**Total Admission Events**	**Admissions Followed by Positive Swabs**	**Percentage**
Tropical	Site A	−12.354860, 130.882845	25	1107	71	6.4%
Tropical	Site B	−16.912178, 145.768408	10	2818	149	5.3%
Tropical	Site C	−19.320384, 146.762324	3	2937	202	6.9%
Tropical	Site D	−21.145531, 149.155603	7	1767	97	5.5%
Subtropical	Site E	−27.227687, 153.104899	13	2942	84	2.9%
Subtropical	Site F	−27.389547, 153.022753	24	7536	264	3.5%

## Appendix C

### Profile of Bacteria Cultured Within 12 Months Following Lower Limb Orthopaedic Procedures in Australian Tropical and Subtropical Hospitals

**Group**	**Species**	**Total (*n* = 867)**	**Tropical (*n* = 519)**	**Sub-tropical (*n* = 348)**	***p***
Staphylococcus	Total	527 (60.8%)	320 (61.7%)	270 (59.5%)	0.52
	*S. aureus*	426 (80.8%)	259 (80.9%)	167 (80.7%)	0.94
	MRSA	101 (19.2%)	61 (19.1%)	40 (19.3%)	
	MDR *S. aureus*	166 (31.5%)	115 (35.9%)	51 (24.6%)	0.006
CONS	Total	47 (5.4%)	24 (4.6%)	23 (6.6%)	0.21
	*S. capitis*	7 (14.9%)	3 (12.5%)	4 (17.4%)	0.49
	*S. epidermidis*	32 (68.1%)	18 (75.0%)	14 (60.9%)	
	*S. lugdunensis*	5 (10.6%)	1 (4.2%)	4 (17.4%)	
	*S. haemolyticus*	3 (6.4%)	2 (8.3%)	1 (4.3%)	
	MDR CONS	31 (66.0%)	17 (70.8%)	14 (60.9%)	0.47
Enterococcus	Total	59 (6.8%)	32 (6.2%)	27 (7.8%)	0.36
	*E. faecalis*	50 (84.7%)	30 (93.8%)	20 (74.1%)	0.066
	*E. faecium*	9 (15.3%)	2 (6.3%)	7 (25.9%)	
	MDR Enterococcus	22 (37.3%)	12 (37.5%)	10 (37.0%)	0.97
Streptococcus	Total	82 (9.5%)	57 (11.0%)	25 (7.2%)	0.061
	Group A	41 (50.0%)	32 (56.1%)	9 (36.0%)	0.010
	Group B	13 (15.9%)	6 (10.5%)	7 (28.0%)	
	Group C	16 (19.5%)	14 (24.6%)	2 (8.0%)	
	Group G	9 (11.0%)	4 (7.0%)	5 (20.0%)	
	Peptostreptococcus	1 (1.2%)	0	1 (4.0%)	
	Pneumonia Group	1 (1.2%)	1 (1.8%)	0	
	Viridans Group	1 (1.2%)	0	1 (4.0%)	
	MDR Streptococcus	0			
Enterobacteriaceae	Total	129 (14.9%)	67 (12.9%)	62 (17.8%)	0.047
	*M. morganii*	11 (8.5%)	5 (7.5%)	6 (9.7%)	0.67
	*P. mirabilis*	13 (10.1%)	7 (10.4%)	6 (9.7%)	
	*E. coli*	20 (15.5%)	11 (16.4%)	9 (14.5%)	
	*K. pneumoniae*	22 (17.1%)	8 (11.9%)	14 (22.6%)	
	*S. marcescens*	27 (20.9%)	16 (23.9%)	11 (17.7%)	
	*E. cloacae*	36 (27.9%)	20 (29.9%)	16 (25.8%)	
	MDR Enterobacteriaceae	50 (38.8%)	26 (38.8%)	24 (38.7%)	0.99
Non-fermenters	Total	224 (25.8%)	149 (28.7%)	75 (21.6%)	0.018
	*A. baumannii*	12 (5.4%)	11 (7.4%)	1 (1.3%)	0.065
	*P. aeruginosa*	212 (94.6%)	138 (92.6%)	74 (98.7%)	
	MDR Nonfermenters	18 (8.0%)	17 (11.4%)	1 (1.3%)	0.009

## Appendix D

### Bacterial Species by Season in a Subtropical Location

**Bacterial Species**	**Overall**	**Sub-Tropical**	**Subtropical**				
			**Spring**	**Summer**	**Autumn**	**Winter**	***p***
	*n*	*n*	*n* (%)	*n* (%)	*n* (%)	*n* (%)	
Staphylococcus	527	207	43 (60.6%)	50 (56.2%)	62 (60.2%)	52 (61.2%)	0.91
MDR *S. aureus*		51	7 (16.3%)	12 (24.0%)	14 (22.6%)	18 (34.6%)	0.21
CONS	47	23	5 (7.0%)	3 (3.4%)	12 (11.7%)	3 (3.5%)	0.069
MDR CONS		14	4 (80.0%)	2 (66.7%)	6 (50.0%)	2 (66.7%)	0.75
Enterococcus	59	27	6 (8.5%)	4 (4.5%)	13 (12.6%)	4 (4.7%)	0.12
MDR Enteroccoccus		10	1 (16.7%)	2 (50.0%)	5 (38.5%)	2 (50.0%)	0.67
Streptococcus	82	25	7 (9.9%)	4 (4.5%)	6 (5.8%)	8 (9.4%)	0.45
MDR Streptococcus		0	*n*/a	*n*/a	*n*/a	*n*/a	*n*/a
Enterobacteriaceae	129	62	11 (15.5%)	23 (25.8%)	12 (11.7%)	16 (18.8%)	0.075
MDR Enterobacteriaceae		24	5 (45.5%)	8 (34.8%)	8 (66.7%)	3 (18.3%)	0.072
Non-fermenters	224	75	12 (16.9%)	23 (25.8%)	22 (21.4%)	18 (21.2%)	0.60
MDR Non-fermenters		1	1 (8.3%)	0 (0.0%)	0 (0.0%)	0 (0.0%)	0.16

## Appendix E

### Bacterial Species by Season in a Tropical Location

**Bacterial Species**	**Overall**	**Tropical**	**Tropical**				
			**Spring**	**Summer**	**Autumn**	**Winter**	***p***
	*n*	*n*	*n* (%)	*n* (%)	*n* (%)	*n* (%)	
Staphylococcus	527	320	73 (60.3%)	85 (66.4%)	96 (63.6%)	66 (55.5%)	0.32
MDR *S. aureus*		115	29 (39.7%)	26 (30.6%)	37 (38.5%)	23 (34.8%)	0.61
CONS	47	24	7 (5.8%)	2 (1.6%)	8 (5.3%)	7 (5.9%)	0.30
MDR CONS		17	4 (57.1%)	1 (50.0%)	5 (62.5%)	7 (100.0%)	0.21
Enterococcus	59	32	10 (8.3%)	6 (4.7%)	9 (6.0%)	7 (5.9%)	0.70
MDR Enteroccoccus		12	5 (50.0%)	1 (16.7%)	2 (22.2%)	4 (57.1%)	0.31
Streptococcus	82	57	16 (13.2%)	11 (8.6%)	11 (7.3%)	19 (16.0%)	0.090
MDR Streptococcus		0	*n*/a	*n*/a	*n*/a	*n*/a	*n*/a
Enterobacteriaceae	129	67	18 (14.9%)	21 (16.4%)	11 (7.3%)	17 (14.3%)	0.10
MDR Enterobacteriaceae		26	7 (38.9%)	8 (38.1%)	4 (36.4%)	7 (41.2%)	1.00
Nonfermenters	224	149	39 (32.2%)	39 (30.5%)	45 (29.8%)	26 (21.8%)	0.29
MDR Nonfermenters		17	5 (12.8%)	6 (15.4%)	4 (8.9%)	2 (7.7%)	0.73
